# Digital health technologies to transform women’s health innovation and inclusive research

**DOI:** 10.1136/bmj-2025-085682

**Published:** 2025-10-10

**Authors:** Bola Grace, Lauren A Wise, Marzena Nieroda, Jennifer Egbunike, Nafisat O Usman

**Affiliations:** 1 UCL EGA Institute for Women’s Health, University College London, London, UK; 2 Department of Epidemiology, Boston University School of Public Health, Boston, USA; 3 UCL Global Business School for Health, University College London, London, UK; 4 Department of Healthcare Management, College of Business, University of Doha for Science and Technology, Doha, Qatar; 5 Department of Community Medicine, Kaduna State University, Nigeria

## Abstract

**Bola Grace and colleagues** argue that using digital health technologies ethically can increase the scope and scale of research and connect systems to improve women’s health

Despite comprising over half the global population, women bear a disproportionate burden of adverse health outcomes with unique challenges.^1^ Historically, women were routinely excluded from clinical trials because of concerns about risks during pregnancy, fluctuating hormones, and a bias that viewed men’s bodies as the norm for medical research.^2^ Recently, there has been a gradual increase in the inclusion of women in clinical research; however, across a broader range of health conditions there is still room for improvement. Barriers faced by women in research participation include access, education, low incentives, digital literacy, transportation, time commitment, and research design.^3^ Addressing factors that influence inequities in women’s health requires innovation, not only in treatments and technologies but also in research designs and methodologies. Digital health technologies (DHTs) —defined as “systems that use computing platforms, connectivity, software, or sensors for health care and related uses”^4^—have facilitated the inclusion of diverse populations throughout the research lifecycle by improving accessibility, engagement, efficiency, and personalisation of interventions. In this article, part of a BMJ Collection on Women’s Health Innovation (www.bmj.com/collections/womens-health-innovation), we describe how DHTs, together with inclusive designs across the research lifecycle, can transform research from an extractive to a participatory process driving inclusion in scope, scale, and systems for improving women’s health.

## Emphasising inclusive research methods for innovation

Social determinants of health refer to “the conditions in which people are born, grow, live, work and age, and people’s access to power, money and resources.”^5^ Historically, the approaches used in health research have often failed women through exclusion largely based on social determinants of health. Moving beyond these historical limitations requires a deliberate effort to adopt and integrate innovative approaches specifically designed to be inclusive, participatory, and sensitive to the nuances of sex, gender, and the diverse experiences of women across their life course.

**Fig 1:**
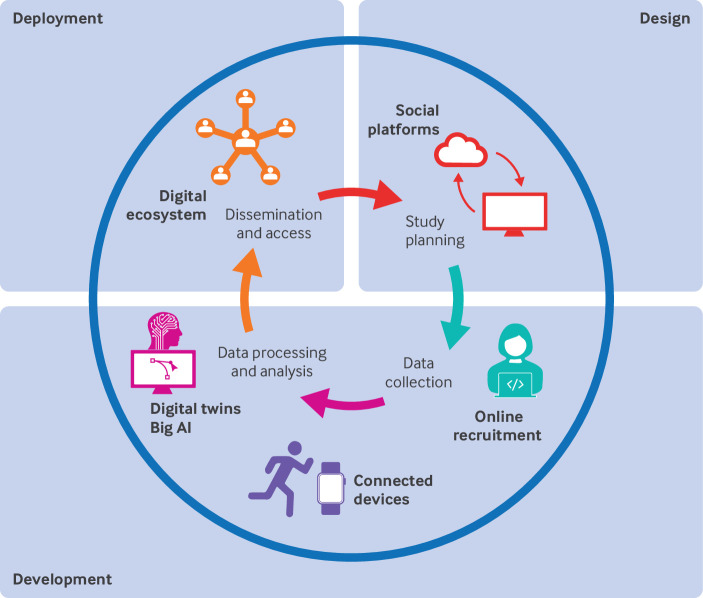
Example of use of digital health technologies for inclusive innovation across the research lifecycle

Inclusive innovation can be defined as the involvement of intended end users in the design, development, and deployment of solutions to drive improvement.^6^ Iterative implementation can be mapped throughout the research lifecycle (fig 1) in a way that is contextually tailored, with consideration given to individual characteristics, place based approaches, and accessibility, not only in terms of physical proximity but also in affordability, appropriateness, and ease of use.

## Digital health technologies driving inclusive innovation in practice

DHTs offer powerful solutions for dealing with longstanding inequities in women’s health research, by shifting power and promoting participation that centres women’s voices and experiences in the design, development, and deployment of solutions intended for them; thus enabling women to participate actively in their own healthcare by improving accessibility, engagement, efficiency, and personalisation. The E3 framework provides a classification for technology maturity and adoption.^6^ Established technologies such as telehealth platforms, mobile health (mHealth), and web based applications

have reduced access barriers, thereby increasing reach and scope, especially in low income settings.

Evolving technologies, such as connected devices and wearable sensors, enable real time monitoring, passive data collection, and personalised, quantifiable objective evidence. These technologies, including the application of artificial intelligence (AI) and machine learning (ML), enable the analysis of large datasets to deliver insights and gather real world evidence at greater pace and scale. Emerging technologies, such as digital twins, big AI, and digital ecosystems, hold promise for digital futures in women’s health providing a connected systemised approach to inclusive research designs, by enabling predictive analysis, personalised treatment, and optimised care pathways. This article provides an analysis of the use of DHT in women’s health across various settings to increase scope, scale, and systems for inclusion (table 1), considering implementation challenges.

**Table 1:** Examples of how technology can increase scope, scale, and systems for women’s inclusion

Inclusive innovation/research phase	Established technologies (increased reach and scope)					Evolving technologies (increased scale with larger datasets)			Emerging technologies (increased connected systems for data aggregation and networks)	
	Web platforms	mHealth	Telehealth			Connected devices	AI and ML		Digital twins and big AI	Digital ecosystems	
**Design**											
Planning	Review of social media platform for research planning		Web based recruitment and online questionnaire administration						Patient specific digital models of cervix, uterus, placenta	Connected datasets	
**Development**											
Execution		Text messages for information delivery asynchronous audiovisual digital media				Electronic collection of users’ reported data	AI supported screening			Connected endometriosis symptom tracking	
Analysis						Analysis of digital biomarkers and digitally derived measures	Data analytics large datasets			Collaborative neuroimaging platform	
**Development**											
Dissemination, exploitation	Interactive contraception choices platform	Information on contraception, sexually transmitted infections, nutrition, health awareness	Pregnancy planning web and mobile application			Personalised cycle tracking system with real world evidence	AI driven multi centre mammography screening		Digital twins for preventing pregnancy complications and adverse maternal outcomes	Optimised care pathways for endometriosis. Federated learning for privacy, security, and interoperability	

## Expanding scope through established technologies

Technology continues to facilitate inclusion of women throughout the research lifecycle. Teleconsultations, virtual focus groups, and digitally informed consent processes can be beneficial for women with caregiving responsibilities, mobility challenges, those in rural areas, or those from minority ethnic groups, all of whom have traditionally been excluded from trials. The covid-19 pandemic accelerated the adoption of telehealth, increasing opportunities for decentralised, remote research.^7^ Innovative study planning and recruitment strategies using digital tools and social media improve engagement of multiethnic groups.^8^ Mobile phone use is high across all demographics, including underserved populations.^9^ There has been a dramatic increase in use of mobile applications for menstrual cycle tracking and fertility symptom recording. A recent study^10^ estimated over 250 million downloads of menstrual tracking apps worldwide. Web platforms, such as the contraception choices interactive platform, help women to make informed choices about contraception.^11^ Additionally, web based digital platforms can be co-designed with users to improve relevance, trust, and ultimately usage.

A review of gender transformative mHealth approaches in maternal health in sub-Saharan Africa^12^ assessed gender blind/gender aware initiatives and gender based health inequities. Findings showed that DHTs are being used to tackle barriers to women’s access, supporting the target in sustainable development goal 5 of ensuring universal access to sexual and reproductive health and reproductive rights. The most common mHealth delivery systems were text messages and asynchronous audiovisual digital media. These messages focused on maternal health services to optimise contraceptive use, reduce unintended pregnancies, manage sexually transmitted infections (STIs), improve postpartum breastfeeding and nutrition, and increase access to skilled healthcare professionals during pregnancy, childbirth, and postpartum. These interventions yielded mostly positive results; however, a notable limitation was poor access to mobile phones among end users in rural communities. Research has also highlighted the lack of adaptation of mHealth apps to meet the needs of sub-Saharan African multicultural, ethnically diverse, and religious population groups.^13^

Another example comes from a North American preconception cohort study. Among participants randomised to receive a premium subscription to a fertility tracking app, adherence (that is, app use) was substantially higher among low income participants and participants of colour relative to other subgroups.^14^ In per protocol analyses, adherence was associated with faster time to pregnancy among those with ≤12 years of education but not other education subgroups. This research indicates, firstly, that offering digital technology to marginalised women can increase inclusion in fertility research (eg, traditionally under-represented participants were more likely to use technology when offered); and secondly, that digital technology use can improve fertility among more marginalised populations (eg, app users with lower education had a faster time to pregnancy). Thus, inclusion and innovation through DHT use can directly improve women’s health.

## Machine learning and AI’s potential for larger scale research

ML and AI models are increasingly applied in the analysis of large scale women’s health data.^15^ When designed with equity in mind, AI tools can help identify underserved subgroups, tailor interventions, and locate diagnostic blind spots. Digitally derived measures from the use of tools such as wearables and connected devices, based on user centred design, have the potential to transform inclusive personalised healthcare for women, providing real world data and evidence.

The lack of inclusive research has left generations of women with little knowledge of their own bodies. Digitally derived biomarkers from connected devices provide real world insights into women’s health. In their analysis of 75 981 anonymised menstrual cycles, as represented by digitally derived biomarkers, researchers found that only 12.4% of users had a 28 day cycle.^16^ Most participants (87%) had cycle lengths between 23 and 35 days, with the most common day of ovulation being day 15, compared with the traditional notion of day 14. Researchers found considerable inter- and intra-individual variability in cycle length. These findings, based on real world evidence from connected devices, highlight the disparity between textbook perception of menstrual health and what women perceive to be “normal.” A limitation was a lack of sociodemographic information owing to privacy requirements for anonymisation. Nevertheless, this inclusive approach to research provides personalised insights as well as aggregate data on biological variation within populations.

A Swedish multisite randomised controlled trial^17^ enrolled 80 033 women aged 40–80 years to assess the clinical safety of AI to support mammography screening compared with standard double reading by radiologists. Participants were randomised to AI supported screening or standard screening. Among 39 996 women in the AI group, 244 cancers were detected versus 203 among 40 024 women in the control group. Cancer detection rates were slightly higher in the AI group (6.1/1000 *v* 5.1/1000), meeting the predefined safety threshold, with similar recall and false positive rates between groups. Notably, AI reduced screen reading workload by 44.3%. Findings suggest that AI supported screening maintains clinical safety while lowering radiologists’ workload, allowing for broader screening of women.

Despite AI’s promise in solving important issues in women’s health, these systems can induce biases if they are trained on non-representative datasets.^18^ AI’s potential is best realised when datasets represent the full diversity of the target population, are stratified by relevant sociodemographic factors, and have transparent data pipelines.

## Accessible research systems

With the continued adoption of emerging technologies, datasets from mobile applications, wearable sensors, imaging, and health records can be combined for individualised data driven treatments, interventions, and personalised services. Accessible research ecosystems also entail understanding the diverse meanings individuals assign to their health behaviours and recognising how their surrounding environments can facilitate related activities.^19^

Endometriosis is a common chronic pain condition with no known cure and limited treatment options. Researchers investigated how traditional data collection through daily diaries and medical histories, coupled with data from mHealth apps and wearables, can provide personalised self-treatment and digital self-management tools to increase quality of life and tailor healthcare for patients with endometriosis.^20^

A digital twin is a virtual representation of a physical object, system, or process, mirroring a real world counterpart.^21^ The rigour of digital twins can be combined with ML’s speed and flexibility for a hybrid model: “big AI.”^22^ Although researchers are still far from implementing a fully featured human digital twin, advances have been made in creating computational models for use in research. For example, researchers have devised techniques to extract minimal measurements of the uterus and cervix from 2D ultrasound images, enabling the creation of patient specific 3D models that simulate mechanical changes in the uterus and cervix during pregnancy, offering a tool for predicting preterm birth, while computation models of the placenta are being used to understand pre-eclampsia and fetal growth restriction.^23^

Despite over 50 000 human brain imaging studies being published to date, <0.5% consider women’s health, even though women constitute 70% of cases of Alzheimer’s and 65% of cases of depression.^24^ Addressing this gap, the brain imaging consortium pioneered a system for organising neuroimaging and behavioural data for easy sharing and reuse, advancing open science and promoting gender inclusive neuroscience.^25^ This large scale data sharing initiative is critical, as women’s health research has historically focused on reproductive conditions, overlooking sex specific differences in the presentation and treatment of neurological, cardiovascular, and respiratory diseases.

The future of applied health data science is increasingly aligned with federated learning, a ML technique where multiple devices or entities collaborate to train a shared model without direct exchange of raw data.^26^ Federated learning promises to overcome privacy, security, and interoperability challenges inherent in centralised AI models. It allows AI systems to be trained collaboratively across multiple institutions without moving sensitive data, ensuring privacy by design. Beyond privacy protection, federated learning could support ecosystem-wide research collaboration by connecting health, social, and environmental data across sectors, enabling more holistic, person centred support.^25^

## Challenges and considerations

The potential of DHTs to enhance scope, scale, and systems for improving women’s health is evident. Despite the promise, widespread and equitable implementation of DHTs for inclusive innovation and research faces several challenges; realising the potential requires confronting the digital gender gap.

Women often face distinct barriers related to cost, connectivity, digital skills, data privacy, and safety concerns online,^27^ potentially hindering adoption of DHT. For example, the Dobbs decision in June 2022 to remove federal level protections for abortion influenced the degree to which US research participants felt comfortable sharing their DHT data.^28^ In states with banned or restricted abortion rights, PRESTO documented a 27 percentage point reduction in fertility app use comparing post-Dobbs (June-November 2022) versus pre-Dobbs (February-June 2022) periods.^28^ Thus, government policy can have profound effects on women’s engagement with DHTs. The environmental effects of digital innovation in healthcare also continue to be of concern,^29^ and specific AI technologies such as large language models and chatbots may lack the empathy crucial for sensitive health discussions. Digital innovation must therefore integrate solutions with targeted strategies for inclusion, user centricity, and robust data governance frameworks that prioritise ethical principles, build trust, and include women in DHT leadership roles.^27^

The “digital divide,” highlighting inequalities in access to devices, reliable internet, and digital literacy, risks exacerbating existing health inequities.^30^ Furthermore, participation is crucial for effectiveness in low and middle income countries. Funders and policymakers should explicitly support inclusive methods, prioritise equitable AI by ensuring diverse population representation in datasets, adopt explainable AI technologies, and engage stakeholders in participatory decision making.^31^

Many digital solutions lack rigorous evidence of effectiveness or adherence to quality and safety standards, and in many instances, health technologies aimed at women have been shown to use feminist narratives to promote non-evidenced based interventions,^32^ under the guise of “empowering” women. This begs the question: “who holds the power in empowerment?” Technology has its limits; therefore, the ultimate focus of any technology driven improvement must always be the individuals it aims to serve. There is a growing body of evidence of technology facilitated violence against women.^33^ Understanding what works, for whom, and under what conditions is therefore critical for directing investments and policies that truly empower women.

The idea of inclusive research is not without criticisms, and suggestions have been made that “calls for inclusive research prioritise ideology over scientific rigour,” with the potential to complicate recruitment, increase risks of type II errors, and introduce confounding.^34^ Nevertheless, despite policy efforts for inclusion in clinical research, sex disaggregated analyses remain scarce, and women of colour are particularly under-represented.^35^ Traditional approaches often examine factors in isolation, yet women’s health is influenced by the dynamic interaction of biological, social, economic, and environmental determinants, as well as systemic issues including sexism and racism.^27^

These interconnected social determinants of health are often overlooked in siloed research, resulting in policies and interventions that do not fully tackle the complex realities of women’s health needs. Many digital health promotion strategies disproportionately emphasise individual responsibility for health, often neglecting the wider social, cultural, and political contexts that shape technology’s use,^31^ ^36^ with digital inclusion being considered a social determinant of health .^37^

A crucial consideration for inclusive research is that the current landscape of DHT in women’s health highlights a persistent focus on childbearing within the range of reproductive health intentions,^38^ and in wider women’s health, a focus on reproduction and pregnancy. While these remain critical areas of women’s health research, this concentration mirrors the historical “bikini medicine” bias,^39^ neglecting the potential of DHTs to consider the full range of women’s health needs across their entire lifespan, including cardiovascular and bone health, neurology, cancer prevention, and mental health.

## Future directions

As women’s health continues to gain attention on the global agenda, inclusion should become a foundational principle. This is even more pertinent in the changing political landscape that continues to threaten diversity, equity, and inclusion.^40^ Table 2 provides recommendations for accelerating women’s inclusion. Through concerted efforts by various stakeholders, DHTs can be optimised to increase inclusion of women in research trials, tackle systemic barriers, advance gender equity in research, and support the development of more effective health solutions tailored to women’s needs.

**Table 2:** Recommendations for accelerating inclusion in women’s health across scope, scale, and systems through digital technologies

Stakeholder group	Scope	Scale	Systems
Researchers, academic	Leverage recruitment strategies that actively engage women (eg, through social platforms, female led groups, or partnering with women’s healthcare providers, clinics, and advocacy groups). Ensure study designs consider gender specific factors that might affect participation and outcomes	Use digital health technologies to offer flexible participation options (eg, remote monitoring and virtual consultations), making it easier for women to integrate trials into their routines	Democratisation of research. Use digital platforms for open data sharing to allow researchers worldwide to access and collaborate on health data, fostering innovation and inclusivity. Implement hybrid research models that combine traditional trials with real world data collection through digital means, broadening participant demographics and scenarios
Innovators, industry	Collaborate with researchers and women’s health organisations to ensure platforms address unique health concerns and are accessible to various subgroups of women. Promote equitable AI through diverse population representation in datasets and inclusive methodologies, involving stakeholders in participatory decision making	Incorporate data on social determinants of health, which can offer a more comprehensive understanding of health risks and outcomes	Increase use of advanced analytics and artificial intelligence to provide actionable insights from large datasets, enabling more informed decision making in healthcare delivery and policy
Research participants, end users	Provide design feedback and share insights on digital technology experiences, highlighting areas for improvement in accessibility and engagement	Actively participate and advocate for raising awareness about the importance of women’s involvement, encouraging peer engagement and broader community recruitment	Challenge exclusionary practices and demand transparency and accountability (eg, through patient advocacy groups and requesting information on data). Join or create online communities that crowdsource research ideas or promote trial opportunities to under-represented groups
Funders, investors	Sponsor gender sensitive research designs and inclusion policies in grant proposals to further promote female representation	Mandate inclusive metrics and establish funding incentives for projects that focus on women’s health issues or address gender disparities, including women in digital health leadership roles	Fund collaborative ecosystems (eg, public-private partnerships) to bring together multiple stakeholders and fund open source tools that encourage the development of shared, open digital platforms that embed inclusion by design
Policy makers, legislators	Promote widespread access to digital tools via subsidised connectivity and devices, especially in underserved areas	Endorse regulatory frameworks that support flexible and adaptive research methodologies, enabling quicker adaptation to emerging digital health technologies and trends. Promote public facing dashboards for trials and monitoring	Incentivise development of digital health platforms that support women’s health holistically around the life course, physiological pattern, beyond “bikini medicine” to better reflect the complex, interconnected nature of women’s health

**Key messages**

- Although women live longer than men, they spend a greater proportion of their lives in poorer health, with growing inequalities in access to services and health outcomes
- The rapid development of digital health technologies and participatory research models offer new opportunities to reach a broader cross section of the population
- Evolving technologies, such as connected devices, wearable sensors, and artificial intelligence, can increase scale for big data analytics
- Emerging technologies, such as digital twins and a fully connected digital health ecosystem, show potential for predictive analyses and optimised care pathways in women’s health
- To deliver the full promise of digital technologies in transforming women’s health, attention must be paid to associated risks, safety, technology ethics, and unintended consequences

## Notes

Contributors and sources: The authors are a diverse group of experts, bringing multidisciplinary expertise across women’s health, digital health, inclusive innovation, population health, epidemiology, clinical practice as well as academic, clinical, and market research. This article builds on decades of cross sectoral experience in women’s health, digital health, and innovation by BG, who serves as the guarantor. All authors contributed to the review and approved this article.

This article is part of a collection developed in partnership with the Gates Foundation. Open access fees were funded by the Gates Foundation. *The BMJ* commissioned, peer reviewed, edited, and made the decision to publish. Smruti Patel and Jocalyn Clark were the lead editors for *The BMJ*.
